# Complex Pathophysiology of Acute Kidney Injury (AKI) in Aging: Epigenetic Regulation, Matrix Remodeling, and the Healing Effects of H_2_S

**DOI:** 10.3390/biom14091165

**Published:** 2024-09-17

**Authors:** Shreyasi Gupta, Subhadeep Mandal, Kalyan Banerjee, Hebah Almarshood, Sathnur B. Pushpakumar, Utpal Sen

**Affiliations:** 1 Department of Zoology, Trivenidevi Bhalotia College, College Para Rd, Raniganj 713347, West Bengal, India; guptashreyasi2009@gmail.com (S.G.);; 2 Department of Physiology, University of Louisville School of Medicine, Louisville, KY 40202, USA; hebahmohammeda.almarshood@louisville.edu (H.A.);

**Keywords:** aging, acute kidney injury (AKI), miRNAs, epigenetics, H_2_S, ECM, macrophages, MMPs, EMMPRIN, Meprin-A

## Abstract

The kidney is an essential excretory organ that works as a filter of toxins and metabolic by-products of the human body and maintains osmotic pressure throughout life. The kidney undergoes several physiological, morphological, and structural changes with age. As life expectancy in humans increases, cell senescence in renal aging is a growing challenge. Identifying age-related kidney disorders and their cause is one of the contemporary public health challenges. While the structural abnormalities to the extracellular matrix (ECM) occur, in part, due to changes in MMPs, EMMPRIN, and Meprin-A, a variety of epigenetic modifiers, such as DNA methylation, histone alterations, changes in small non-coding RNA, and microRNA (miRNA) expressions are proven to play pivotal roles in renal pathology. An aged kidney is vulnerable to acute injury due to ischemia-reperfusion, toxic medications, altered matrix proteins, systemic hemodynamics, etc., non-coding RNA and miRNAs play an important role in renal homeostasis, and alterations of their expressions can be considered as a good marker for AKI. Other epigenetic changes, such as histone modifications and DNA methylation, are also evident in AKI pathophysiology. The endogenous production of gaseous molecule hydrogen sulfide (H_2_S) was documented in the early 1980s, but its ameliorative effects, especially on kidney injury, still need further research to understand its molecular mode of action in detail. H_2_S donors heal fibrotic kidney tissues, attenuate oxidative stress, apoptosis, inflammation, and GFR, and also modulate the renin–angiotensin–aldosterone system (RAAS). In this review, we discuss the complex pathophysiological interplay in AKI and its available treatments along with future perspectives. The basic role of H_2_S in the kidney has been summarized, and recent references and knowledge gaps are also addressed. Finally, the healing effects of H_2_S in AKI are described with special emphasis on epigenetic regulation and matrix remodeling.

## 1. Introduction

Acute kidney injury (AKI) is a pathophysiological condition that can be diagnosed by elevated creatinine levels and/or decreased urine output [1]. In developed countries, the incidence of severe AKI is almost 3.2 to 9.6% of total hospitalized patients, whereas in developing countries, AKI mortality rates are still as high as 50–60% [2] and need much more attention. AKI causes approximately 2 million fatalities annually worldwide [3,4] and affects up to 50% of critically ill intensive care unit (ICU) patients as their severity increases mortality rates [5,6]. Patients who are old or have a prior disease history, such as chronic kidney disease (CKD), diabetes, cancer, etc., are more vulnerable to form AKI. However, several molecular mechanisms underlying different stages of AKI pathogenesis are still a matter of conjecture and very patient-specific. It is therefore important to identify novel treatment targets and early diagnostic indicators to reduce the mortality rate. One of the major causes of AKI is ischemia-reperfusion (I/R) damage [7,8]. I/R causes inflammatory cells like macrophages to infiltrate within kidney tissue from blood vessels and induce an immunological inflammatory response that leads to the onset of AKI [9]. Moreover, patients who have survived an AKI are more likely to develop CKD [10,11,12].

Age plays a crucial role in kidney health. The replenishment of renal parenchyma significantly decreases in geriatric conditions, and fibrosis takes place due to excessive extracellular matrix (ECM) production in the kidney interstitium [13]. Declined renal function with age is multidimensional and undoubtedly leads to susceptibility to AKI [14], especially nephrotoxic AKI and I/R AKI, and also a declined GFR and healthy nephron number, and tubular epithelial cells [15,16,17]. Cohort studies showed that dialysis is required in AKI in elderly patients with a significantly higher percentage [18,19].

**Figure 1:**
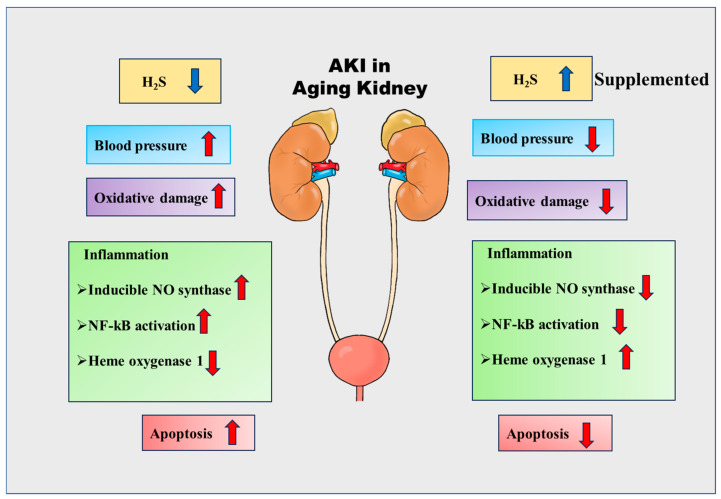
Effect of H_2_S in pathophysiological AKI: In the absence or at the low physiological level of H_2_S blood pressure [22,23], oxidative damage [24,25,26], inducible NO synthase [27,28], NF-kB activation [28,42], and apoptosis increase [29,30] and Heme oxygenase 1 decreases [28,31,32] in aging AKI. When H_2_S is supplemented, these changes are reversed (shown on the right side of the schematic images).

Hydrogen sulfide (H_2_S) is one of the endogenous gasotransmitters and is involved in a variety of biological processes [20,21]. The study suggests that H_2_S donors can regulate the rate of blood pressure, oxidative damage, inducible NO synthase, NF-kβ activation, and apoptosis in damaged and aged kidneys [22,23,24,25,26,27,28,29,30,31,32] (Figure 1). The possible healing functions of H_2_S in AKI, e.g., regulating renin release, oxygen sensing, salt absorption, and the glomerular filtration rate (GFR), in association with aging, have been evident in several research [33,34,35,36,37,38,39,40]. H_2_S donors are reported to ameliorate aging-induced kidney changes probably by inhibiting the IR/IRS-2-Akt-mTORC1 signaling pathways, leading to matrix protein synthesis [41].

In this review, we discuss the recent advances in experimental as well as preclinical and clinical research in renal aging and AKI with special references to different epigenetic regulations, such as DNA methylation, histone alterations, non-coding RNA regulation, and/or changes in microRNA. We also discuss how the H_2_S level significantly facilitates the production of the renal matrix in association with EMMPRIN, Meprin-A, and MMPs and helps healing. In the final part, current treatment modalities and therapeutic options with future possibilities and challenges are summarized.

## 2. Production and Detection of H_2_S

Hydrogen sulfide (H_2_S) altered N-methyl-D-aspartate (NMDA) receptor-mediated functions in the hippocampus, and thus, the physiological significance of H_2_S first became apparent in 1996 [43]. Endogenous H_2_S serves several pleiotropic functions within the cardiovascular system, kidneys, liver, pancreas, gastrointestinal system, brain, and reproductive system, including others [34,44,45,46,47,48]. The enzymes responsible for H_2_S synthesis are the following: cystathionine β-synthase (CBS) [49], cystathionine γ-lyase (CSE) [50,51], and 3-mercaptopyruvate sulfurtransferase (3-MST) [52]. H_2_S is present in blood from the nanomolar to micromolar range and thus can travel to different tissues as per requirement [53], although it was further assumed that the micromolar range is a bit of an overestimation due to the limitations in the detection methods [54]. However, regardless of its concentration in the blood, plasma, or tissue, it is a well-recognized gaseous transmitter that has immense physiological effects [54].

Kidneys are a major producer of H_2_S [38,55], in addition to the liver, brain, heart, skeletal muscle, and other major tissues [39]. Under physiological conditions, all three enzymes, i.e., CBS, CSE, and 3-MST, are involved in H_2_S production, with CBS and CSE being the major contributors in the kidney [38,39]. It is reported that 3-MST acts in conjunction with cysteine aminotransferase [56]. In the mitochondria, H_2_S is produced by the reduction of thiosulfate catalyzed by thiosulfate transferase (TST) [56]. Biochemical studies have shown that kidneys are a rich source of enzymatic generation of H_2_S by all five enzymes [56,57].

**Figure 2:**
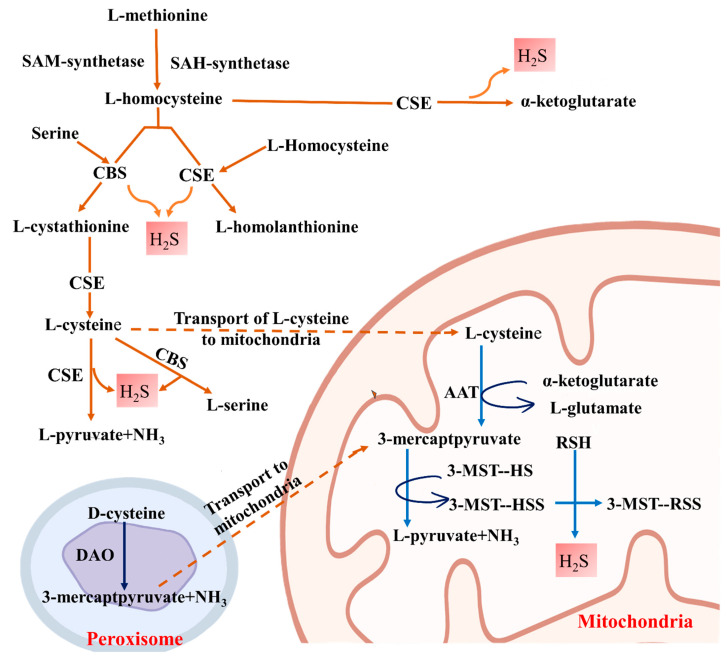
H_2_S production pathway in a cell. In cytosol, L-Methionine is converted into L-homocysteine by S-Adenosylmethionine synthase (SAM) and S-Adenosyl homocysteine hydrolase (SAH). L-homocysteine is condensed with serine by cystathionine β-synthase (CBS) to generate L-cystathionine, which is converted to L-cysteine by cystathionine γ-lyase (CSE). L-cysteine can be used as a substrate by both CBS and CSE to produce hydrogen sulfide (H_2_S). CSE catalyzes H_2_S production by two pathways; one is the conversion of L-homocysteine to L-homolanthionine. Additionally, CSE also catalyzes H_2_S production by the conversion of L-homocysteine to α-ketoglutarate. L-cysteine is transported into mitochondria, and it is converted by aspartate aminotransferase (AAT) in the presence of α-ketoglutarate to form 3-mercaptopyruvate (3-MP) and L-glutamate. Another enzyme, 3-mercaptopyruvate sulfurtransferase (3-MST), contributes to the endogenous H_2_S production in the mitochondria by using 3-mercaptopyruvate (3-MP) as a substrate. Peroxisome resident D-amino acid oxidase (DAO) catalyzes D-cysteine into 3-mercaptopyruvate (3-MP), which is then transported into mitochondria and utilized by 3-MST for the production of H_2_S.

H_2_S is produced enzymatically by two different mechanisms, including pyridoxal 5′-phosphate-dependent (CBS and CSE) and pyridoxal 5′-phosphate-independent (3-MST) systems [21,58,59]. In renal tissue, it has been noted that CBS primarily localizes to the proximal convoluted tubule in the outer cortex [60,61]. According to the reports, the CSE enzyme is localized in the glomerulus and tubulointerstitial region [62]. In addition, 3-MST is localized in the proximal tubular epithelium [63]. CBS mediates the conversion of l-homocysteine (Hcy) and L-Serine to produce cystathionine, which is then converted by CSE into l-cysteine [64,65]. Furthermore, Hcy is converted to homolanthionine by CSE, which catalyzes the formation of H_2_S [66]. Finally, aspartate aminotransferase uses α-ketoglutarate to convert cysteine into 3-mercaptopyruvate and glutamate. In the presence of a reductant, 3-mercaptopyruvate sulfur transferase (3-MST), an additional enzyme, uses 3-mercaptopyruvate as its substrate to contribute to the endogenous synthesis of H_2_S in the mitochondria [52,67]. Further research revealed that 3-MST and D-amino acid oxidase (DAO) are involved in the production of H_2_S from D-cysteine [68]. D-serine is thought to create more H_2_S in the kidney than L-serine [68]. A schematic diagram is shown in Figure 2.

The detection of H_2_S levels in blood or tissue requires an accurate, sensitive, and analytical method [69,70]. Researchers have developed methods for detecting H_2_S, such as gas chromatography, electrochemistry, high-performance liquid chromatography, inductively coupled plasma-atomic emission, UV–visible absorption spectrometry, and fluorescence spectrometry [69,71,72,73]. Compared to other detection methods, electrochemical sensors offer high sensitivity and convenience in H_2_S detection, while fluorescent and colorimetric sensors provide a fast response time, high efficiency and low cost, visualization, and better in vivo imaging [69,72,74,75,76,77,78]. Combining these advantages could improve H_2_S sensor performance and open up new ways to further explore novel H_2_S chemical sensors with excellent properties. Advanced chemical sensors for a highly sensitive real-time visual detection are a possibility as a result of future developments in nanomaterials, fluorescent dyes, and high-precision detection technologies [69].

## 3. The Function of H_2_S in Kidney

Hydrogen sulfide (H_2_S) plays a crucial role in maintaining physiological homeostasis through various mechanisms [79]. It regulates kinases, ion channels, and transcription factors by S-sulfhydrating cysteine residues. Additionally, H_2_S binds to heme in heme-containing proteins, acts as a free radical scavenger, and donates electrons to the mitochondrial electron transport chain, enhancing mitochondrial ATP production and regulating bioenergetics [36,45,80,81,82]. The functions of H_2_S are tissue-specific and we focused on summarizing its role in the kidney below.

### 3.1. H_2_S Effect on Renal Excretory Function and Water Handling

It has been demonstrated that H_2_S alters cellular function in the kidney in a number of ways, with a range of downstream implications. The intrarenal injection of donor H_2_S, NaHS can raise the glomerular filtration rate (GFR), urinary sodium (Una.V), and potassium excretion (Uk.V) [34,35]. Moreover, the effect is closely mimicked by the infusion of L-cysteine, an H_2_S-generating substrate [34].

In addition, the inhibition of endogenous H_2_S production by aminooxyacetic acid (AOAA) (CBS inhibitor) plus DL-propargylglycine (PAG) (CSE inhibitor) causes GFR, Una.V, and Uk.V to decline. This finding indicates that under physiological conditions, H_2_S controls renal function [39]. Another investigation has established that there is a compensating effect between CBS and CSE on renal control, while neither AOAA nor PAG alone can have any effect on renal function [39,83].

**Figure 3:**
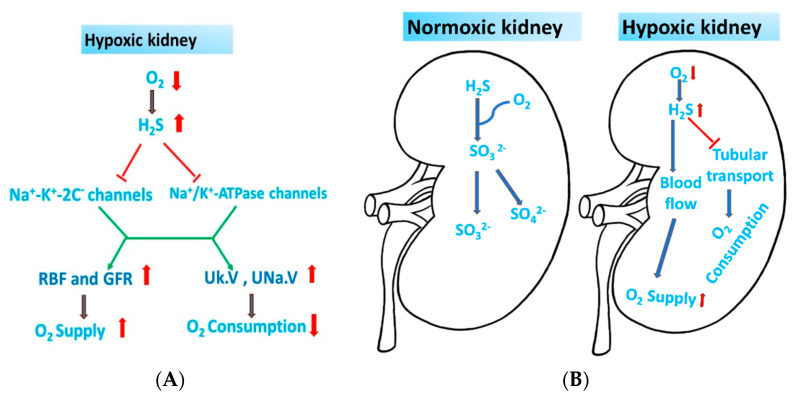
Effects of H_2_S on renal function. (**A**) H_2_S inhibits the activity of tubular Na^+^-K^+^-2C^−^ channels and Na^+^/K^+^-ATPase channels, thereby enhancing renal excretory function, such as RBF and GFR, and the restoration of the oxygen level. (**B**) H_2_S as an O_2_ sensor in the kidney. In normoxic conditions, H_2_S is metabolized into sulfates in the presence of O_2_; in hypoxic conditions, the shortage of O_2_ leads to the accumulation of H_2_S, which helps to restore the O_2_ level by enhancing blood flow and suppressing tubular transport activity in the kidney. H_2_S, hydrogen sulfide; RBF, renal blood flow; GFR, glomerular filtration rate; Una.V, urinary sodium; Uk.V, urinary potassium.

In summary, H_2_S promotes vasodilation and inhibits the activities of Na^+^/K^+^-ATPase (NKA) and the Na^+^-K^+^-Cl^−^-cotransporter (NKCC), which in turn raises renal blood flow (RBF) and GFR and encourages natriuresis (UNa.V) and kaliuresis (Uk.V) [36] (Figure 3A). However, it is still unclear how H_2_S affects the kidney’s ionic exchanger ability.

Several evidence suggest that H_2_S is involved in renal water handling [36,84]. Renal aquaporin 2 (AQP2) expression is upregulated when H_2_S activates the intracellular cyclic adenosine monophosphate (cAMP)/protein kinase A (PKA) signaling pathway [36,85]. This improves urine concentration and lowers urine osmolality in conditions affecting water balance, such as nephrogenic diabetic insipidus [36,84,85].

### 3.2. H_2_S Acts as an O_2_ Sensor in the Kidney

H_2_S serves as an oxygen sensor in the renal medulla during hypoxia, whereupon its production rises, promoting oxygen restoration, raising RBF and GFR, and inhibiting tubular transit [36]. While H_2_S is produced without oxygen, its oxidative metabolism in mitochondria requires oxygen [86]. The availability of O_2_ in renal medulla is lower compared with that in the renal cortex, resulting in a higher abundance of H_2_S in this region [87]. Therefore, the hypoxic environment caused by the low oxygen partial pressure in the renal medulla causes H_2_S to accumulate. This, in turn, increases H_2_S activity, including electron donation for ATP production in the mitochondria, and restores the oxygen balance by increasing the medullary flow and decreasing the tubular Na^+^ transport, which accounts for 60% of renal oxygen consumption [36,83,86,87,88,89,90,91] (Figure 3B). Furthermore, in hypoxic conditions, CBS and CSE can go into mitochondria and induce the synthesis of H_2_S [36,82,92]. However, more research is needed to fully understand the precise processes and subsequent signaling events.

### 3.3. H_2_S Modulates the Renin–Angiotensin–Aldosterone System (RAAS)

RAS activity is determined by the release of renin from juxtaglomerular cells (JG), and intracellular cAMP is known to modulate this process [39,93,94]. The renin–angioten–in–aldosterone system (RAAS) is a complex endocrine system that regulates electrolyte levels and blood volume to maintain blood pressure [95,96,97]. H_2_S has been shown to lower cAMP levels by inhibiting adenylyl cyclase (AC) in a variety of cell types, suggesting that it may control the release of renin, as well as reduce angiotensin II levels [39,98]. According to Lu, Ming et al., the H_2_S donor NaHS prevented and cured hypertension in the two-kidney and one-clip (2K1C) model of renovascular hypertension [98]. On the other hand, in rats that were normal or had one kidney, one clip (1K1C), both of which had normal plasma renin activity, NaHS had no effect on blood pressure or plasma renin activity [36,98]. In other studies, using primary cultures of renin-rich kidney cells, it was shown that treatment with 100 μmol/L of NaHS likewise markedly reduced the level of intracellular cAMP and repressed renin activity [36,98]. Laggner et al. showed that H_2_S directly reduces the activity of the angiotensin-converting enzyme (ACE) in a dose-dependent way [99]. In addition to AC, ROS was recently shown to be a target of H_2_S due to its impact on lowering renin levels in diabetic nephropathy (DN), indicating the involvement of several mechanisms in the process [36,39].

### 3.4. Aging Affects H_2_S Production

Our body naturally produces H_2_S, a gas that plays several roles in physiology, including controlling blood pressure, inflammation, the immune system, energy metabolism, and vascular function, among others [23,100]. Studies have also demonstrated the anti-aging [101] properties of H_2_S by demonstrating its antioxidant, anti-inflammatory [102,103], and cytoprotective [104] effects. One of the main contributing factors to aging and the emergence of age-related diseases is thought to be tissue damage by free radicals. In order to combat oxidative stress and possibly slow down the aging process, H_2_S has been shown to scavenge reactive oxygen species (ROS) [105] and increase the activity of antioxidant enzymes [106]. Pro-inflammatory cytokines, which are proteins that stimulate inflammation, are inhibited by H_2_S, which can thus reduce inflammation [104].

As we age, mitochondrial function declines, which can lead to a number of age-related problems. H_2_S can help to improve mitochondrial function by increasing the production of ATP [107,108]. The effects of aging may differ among tissues. It is known that the cardiovascular system is more susceptible to age-related changes than the liver [109,110]. Although the kidney undergoes structural and functional changes with aging, there are little data regarding the underlying mechanism [111]. However, it is not a surprise that due to age-related changes to the kidney, H_2_S production and signaling may also decline. A reduction in endogenous H_2_S production is reported to accelerate atherosclerosis, an age-dependent disease [112]. In fact, it has been demonstrated that inhibiting CBS expression in human umbilical vein endothelial cells causes premature aging [113]. Further, aging may reduce H_2_S production by causing oxidative damage to CBS/CSE proteins [114]. The above studies provide indirect evidence that a lack of H_2_S is associated with the aging process.

## 4. Epigenetic Regulation of Renal Aging and AKI

AKI may have multiple underlying causes and is linked to intricate pathophysiological processes [115,116,117]. In the last few decades, there has been a rise in the incidence of AKI [115,117]. Mortality rates have declined in critically ill patients with AKI, but mortality rates are still significantly high and increase with AKI severity, specifically in dialysis-requiring AKI [117,118,119,120,121].

The study of heritable mechanisms that regulate gene expression without altering the fundamental nucleotide sequence is known as epigenetics [122,123]. A variety of epigenetic modifiers, such as DNA methylation, histone alterations, non-coding RNA regulation, and/or changes in miRNA expressions are proven to have pivotal roles in molecular alterations during renal aging [124].

### 4.1. DNA Methylation and AKI

DNA methylation happens at the cytosines in CpG dinucleotides to generate 5-methylcytosine (5-mC) [125]; however, it can also occur at a low frequency in non-CpG sites, particularly in embryonic stem cells [126,127], oocytes [128], and brain tissues [129,130]. Sixty to ninety percent of CpG sites in the mammalian genome are methylated [124]. In eukaryotes, DNA methylation occurs only at cytosine residues and involves the covalent addition of a methyl group (CH_3_), which is commonly donated by S-adenosyl-L-methionine (SAM), to the 5-carbon position of the cytosine, creating 5-methylcytosine (5mC) via DNA methyltransferases (DNMTs) [131,132]. Three DNA methyltransferases (DNMTs)—DNMT1, DNMT3a, and DNMT3b—regularly create and maintain DNA methylation patterns in mammals [133,134,135].

In a recent study, Gao et al. found that administering DNA-demethylating agents, SGI-1027 and OLP, effectively alleviated D-gal-induced aging-related structural and functional alteration in mouse kidneys by reducing the DNA methylation of the anti-aging nuclear factor erythroid-derived 2-like 2 (NRF2) and KLOTHO promoter [135,136,137]. According to this study, certain gene expression patterns and genomic DNA methylation may actually have an impact on the aging process of the kidneys. In particular, the kidney aging process is greatly influenced by the dysregulation of DNMT1/3a/3b, and renal aging changes can be lessened by epigenetic intervention using DNA-demethylating agents [133,134,135,136,137]. The current information on DNA methylation changes in age-related kidney diseases is insufficient, necessitating future systematic studies and clinical applications to better understand the molecular mechanisms.

### 4.2. Histone Alterations in AKI

Histone alterations during translation have the ability to control aging and activate or repress gene expression [124,138]. The types of histone modifications include methylation, acetylation, phosphorylation, ubiquitination, ADP ribosylation, and other histone alterations [124]. Particularly, histone methylation and acetylation are among the most studied epigenetic mechanisms in AKI and repair [124,139]. Global aging-related alterations in H3K9me3, H4K20me3, H3K27me3, and H3K9ac levels have been also documented in both in vitro and in vivo studies [124,140].

### 4.3. Non-Coding RNA Regulation in AKI

**Figure 4:**
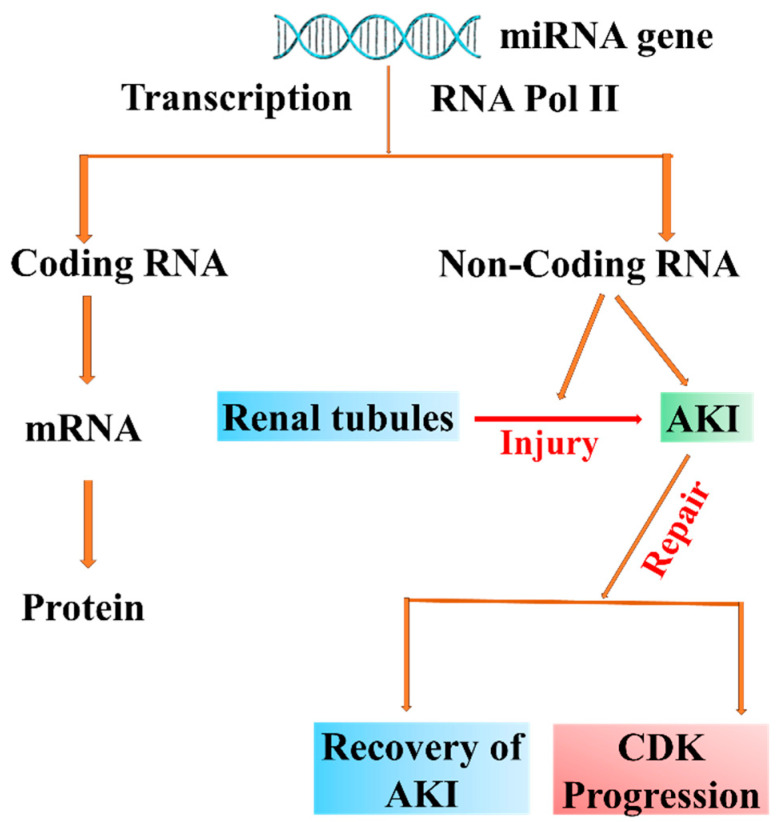
Schematic representation of non-coding RNAs (ncRNAs) that play a role in acute kidney injury (AKI) and subsequent kidney repair. Non-coding RNAs (ncRNAs) are the genome transcripts that function at the RNA level instead of the protein level. AKI, mainly induced by sepsis, renal ischemia-reperfusion, and nephrotoxicity, is pathologically characterized by the injury and death of renal tubular epithelial cells. Complete repair restores the structural integrity and full normal function of the kidney, while maladaptive or incomplete repair leads to chronic pathologies and likely progression to chronic kidney disease (CKD). ncRNAs play regulatory roles in both kidney injury and repair processes, with some being protective and others being pathogenic.

The bulk of the human genome is translated into non-coding RNAs, with less than 2% of it being composed of RNAs having the ability to code for proteins [139]. Non-coding RNAs (ncRNAs) are the genome transcripts that function at the RNA level instead of the protein level, which mainly include microRNAs (miRNAs), long non-coding RNAs (lncRNAs), circular RNAs (circRNAs), small nucleolar RNAs (snoRNAs), and transfer RNAs (tRNAs) [141,142,143,144]. Non-coding RNAs have become significant epigenetic regulators of AKI development and subsequent kidney repair, especially miRNAs and lncRNAs [12,139,145] (Figure 4). Furthermore, non-coding RNAs in circulation may serve as possible biomarkers for AKI [139].

#### 4.3.1. Long Non-Coding RNAs (lncRNA) and AKI

Long non-coding RNAs (lncRNAs) are >200 bp long non-protein-encoding RNAs, which were discovered earlier than miRNA [12,146]. Brannan et al. found the first lncRNA, H19, in 1990, and then a series of lncRNAs was identified [12,147,148]. lncRNAs can attach to histone-modifying complexes to decrease the expression of genes at both the transcriptional and post-transcriptional stages [12,143,149,150]. Nevertheless, there are not enough direct data to draw a conclusion on the role of lncRNAs in kidney healing after AKI; moreover, there is growing proof that lncRNAs play a role in kidney illnesses such as AKI, CKD, kidney transplantation, renal cell cancer, and others [12,139,151].

Using RNA-seq whole-transcriptome profiling reveals that three lncRNAs (MIR210HG, long intergenic non-coding (linc)-ATP13A4–8, and linc-KIAA1737–2) were found in human kidney biopsy samples from kidney transplant patients and were significantly stimulated by hypoxia and cytokines; nevertheless, it is unknown what role these lncRNAs play in AKI and healing [152,153,154].

A different study that employed chromatin immunoprecipitation (ChIP) sequencing of HIF1 and RNA-seq revealed that DARS antisense RNA1 (DARS-AS1) silencing promoted death in cultured renal tubular cells in response to hypoxia, suggesting that DARS-AS1 guards against cell damage brought on by hypoxia [139,153,155].

RANTES, an essential inflammatory mediator in ischemic AKI, is regulated upon the activation of the normal T-cell. The regulation of RANTES synthesis under hypoxic settings by lncRNA-PRINS raises the possibility that lncRNA-PRINS plays a role in the pathophysiology of AKI [139,155].

According to a 2015 study, MALAT1 was the most highly elevated lncRNA in the kidneys of mice that were exposed to inspiratory hypoxia, especially in the proximal tubular cells [139,156]. A more recent study on plasma samples from patients with AKI and biopsy samples from humans with ischemic injury showed the MALAT1 levels to be increased [139,157]. When taken as a whole, these results suggest that lncRNAs might be essential for controlling AKI.

#### 4.3.2. AKI and miRNAs

**Figure 5:**
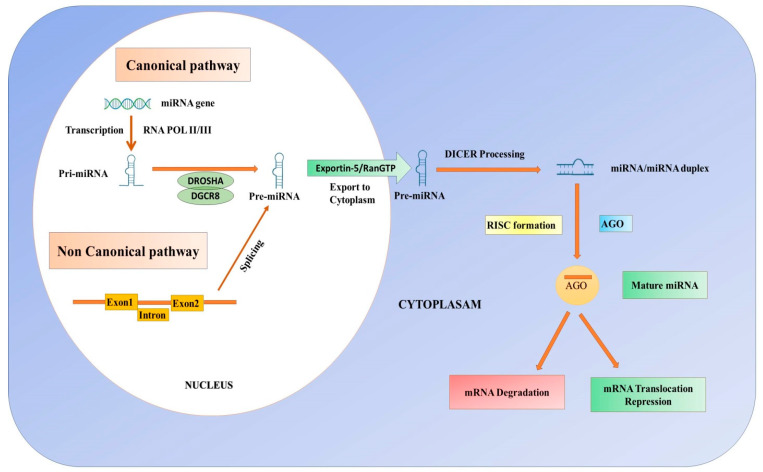
Schematic overview of canonical and non-canonical biogenesis pathways of miRNA: The canonical miRNA pathway produces Pri-miRNA transcripts from miRNA genes by RNA polymerase II, which is processed into pre-miRNAs by the DROSHA/DGCR8 complex. In the non-canonical pathway, pre-miRNAs are directly produced by the splicing of short introns without DROSHA/DGCR8 processing. After transportation to the cytoplasm via Exportin 5, these pre-miRNAs are converted into a double-stranded miRNA/miRNA duplex by DICER. After that, the mature miRNA integrates into RISC. One strand of this duplex is loaded onto AGO and incorporated into the RNA-induced silencing complex (RISC), leading to gene silencing by mRNA degradation and/or translational repression.

MicroRNAs, also known as non-coding single-stranded endogenous miRNAs, have between 19 and 23 nucleotides in length and are involved in post-transcriptional gene silencing in all eukaryotes [158,159,160,161,162,163]. Ambros’s group in 1993 first discovered miRNA in *Caenorhabditis elegans* and showed surprisingly high conservation across species [158,162,163,164]. The biogenesis of miRNAs starts in the nucleus, where the miRNA gene is transcribed by RNA polymerase II, to produce a long primary miRNA [165,166,167] (Figure 5).

A number of conditions, such as ischemia, toxins, sepsis, the obstruction of the urinary tract or the bladder’s outflow, and inflammation, may cause AKI. The dramatic protective effect seen in proximal tubule-specific Dicer knockout mice, where over 80% of miRNAs were reduced, provided the first proof of the crucial function of miRNA in AKI [168]. It is of note that although miRNAs have been reported to play a variety of roles in the kidney, including cellular homeostasis, apoptosis, collagen deposition, and tubular function in I/R-induced AKI, their roles in young vs. old kidney vasculature are still a matter of conjecture. Nevertheless, AKI is commonly seen in the older population. However, the research involving aging animals in this area is limited compared to young animals [169,170,171,172,173,174].

Small non-coding RNAs are known as microRNAs (miRNAs) that control more than 90% of the genes in our body by post-transcriptional regulation. Studies have demonstrated that miRNAs are required for podocyte [175], tubular function [176,177], and renin expression by the juxtaglomerular cells [178].

Wei et al. reported the first instance of miRNAs playing pathogenic roles in AKI and developed a Dicer-knockout mouse model [168]. Proximal tubular cells were precisely eliminated from the Dicer gene, which is required to produce miRNAs. In the renal cortex of these mice, there is a global downregulation of microRNAs. They have normal histology and renal function under controlled conditions but show resistance to the AKI that follows bilateral renal I/R. In comparison to their wild-type counterparts, Dicer-null mice exhibit markedly improved renal function, fewer instances of tissue damage, less tubular apoptosis, and higher survival rates under the latter circumstances [168]. These findings therefore suggest a net pathogenic role for microRNA expression in ischemic AKI. Overall, more than 50 individual microRNAs have now been described as having differential expression in AKI [162].

Many of these microRNAs are involved in experimental and clinical AKI and have been associated with pathways at the molecular level that cause inflammation, apoptosis, and fibrosis. The anti-inflammatory, anti-apoptotic, anti-fibrotic, and pro-angiogenic activities of other AKI-associated microRNAs, on the other hand, might operate as protective mechanisms. Therefore, there are a number of crucial factors to consider when evaluating microRNA patterns in AKI [179].

The miRNA-21 is expressed in the kidneys of mammals and, among others, in heart and lung tissue [180]. In numerous preclinical and clinical I/R research, it has been analyzed and assessed as an important player in tissue protection. For example, it reduces renal I/R damage by preventing apoptosis [181]. Suppressing miRNA-21 expression leads to decreased TNF activity and monocyte chemo-attractant protein-1 (MCP-1), and thereby kidney inflammation [182]. Contrarily, a number of studies have shown that miRNA-21 is differentially released in plasma and/or urine in AKI and is pro-inflammatory. It is often increased in AKI and promotes apoptosis, inflammation, and fibrosis in the kidney [183,184]. Thus, miRNA-21 appears to have a dual function; while it inhibits apoptosis and inflammation to protect against injury, it may also amplify the injury response and encourage fibrosis. According to studies, miRNA-21 prevents apoptosis by upregulating B-cell lymphoma 2 (BCL-2), downregulating the phosphatase and tensin homolog (PTEN), activating the AKT pathway, and downregulating the programmed cell death protein 4 (PDCD4) [185]. It also lowers the levels of the active caspase-3 and caspase-8 proteins [181,186]. The upregulation of miRNA-21 also reduces inflammation by increasing IL-10 and lowering the expression of NF-kB, TNF, interleukin 6 (IL-6), and IL-18 [181]. In animal models of AKI, the experimental upregulation of miRNA-21 offers morphologic and functional renoprotection [181,186,187]. In addition, evidence suggests that the overexpression of miRNA-21 suppresses CSE mRNA and protein in cultured aortic smooth muscle cells and injured carotid arteries, which in turn reduces H_2_S production [188]. Thus, its role on tissue, i.e., whether protective or damaging, appears to be pathology- and severity-dependent. Table 1 summarizes the findings indicating miRNA-21 as a potential biomarker of AKI in animal models and human samples, and Table 2 summarizes other miRNAs that are associated with AKI.

**Table 1:** The miRNA-21 studies that are focused on biomarker potential in AKI in animal models and human samples.

**miRNA-21 as Potential Biomarker in Animal Models of AKI**			
**Sample Type and Status**	**AKI Model**	**Origin of Sample**	**Refs.**
miRNA-21 was upregulated in kidneys after warm ischemia in mice	Unilateral renal ischemia in mice	Kidney tissue	[189]
Urinary miRNA-21 is a marker of hypertensive kidney injury in rats	Hypertensive mice with renal tubular lesion	Urine	[190]
miRNA-21 and the miRNA-17-family are activated after I/R injury in mice	Lethal and sub-lethal ischemia in mice	Kidney tissue	[191]
miRNA-21 upregulated in rat kidneys after I/R injury	I/R injury or gentamicin in rats	Plasma and urine	[192]
miRNA-21 was increased on day 8 after dosing	Cisplatin-induced kidney injury in rat	Urine/Kidney tissue	[193]
miRNA-21 was increased in AKI preceding the increase in blood urea nitrogen and creatinine	Aristolochic acid I (AAI)-induced AKI in rat	Plasma	[194]
**miRNA-21 as Potential Biomarker in Patients with AKI in Human**			
**AKI Model/Population**	**Sample Type**	**Role in AKI**	**Refs.**
Patients with AKI (*n* = 98; 27 kidney transplant recipients with biopsy-proven tubular damage and 71 AKI patients in the intensive care unit (ICU)) and patients without AKI (*n* = 97; 74 healthy volunteers and 23 ICU patients without AKI).	Urine	Biomarker of diagnosis	[195]
120 adult patients undergoing cardiac surgery: 40 non-AKI controls, 39 patients with progressive AKI, and 41 with non-progressive AKI.	Urine and plasma	Biomarker of diagnosis	[196]
Consecutive patients (*n* = 115) undergoing major cardiac surgery.	Plasma	Biomarker of diagnosis	[197]

miRNA-10a is renal tubule-specific and is released from tissues upon injury [162]. Its levels in urine and kidney tissue are elevated and lowered in mouse models of renal I/R injury and streptozocin (STZ)-induced diabetic nephropathy (DN), respectively [198,199]. It is believed that miRNA-10a targets IL-12/IL-23p40 to exert protective effects during damage and a pro-apoptotic BCL2-family protein (BIM) [200]. Among critically ill patients in intensive care units (ICUs), reduced plasma levels of miRNA-10a have been demonstrated to predict AKI [201].

In cisplatin nephrotoxic AKI, to prevent damage and death to tubular cells, it is reported that p53 induces miRNA-34a [202]. The miRNA-107 belongs to the miRNA-15/107 family. Members of this family share the AGCAGC at their 5′ end and are involved in a number of pathways that are important for AKI, such as angiogenesis and the stress response [203]. Additionally, miRNA-107 was discovered to be upregulated in circulating endothelial cells in patients with septic-induced AKI [204].

A study has shown that miRNA-129 is involved in Ang-II-induced renal inflammation through an epigenetic mechanism [205]. In 10–12 weeks old mice undergoing unilateral warm ischemia, global miRNA profiling revealed the upregulation of miRNA-21 and miRNA-20a on day one, and on day three, revealed the upregulation of miRNA-146, -199a-3p, and -214 [189]. Other downregulated miRNAs include miRNAs-192, -194, and -197 [189]. The inhibition of miRNA-24 has been reported to suppress the apoptosis of epithelial and endothelial cells, thereby increasing vascular density and reducing tubular fibrosis [206]. These reports suggest that miRNAs play a great role in I/R-induced AKI; however, the dysregulation of miRNAs and their functional relevance in aging kidneys have yet to be fully deciphered. Although miRNA-194 is differentially expressed in I/R-injured kidneys, its role in H_2_S production remains unknown [189].

The dysregulation of several miRNAs has also been reported in renal diseases, including miRNA-192 involving collagen deposition in DN [207], and the upregulation of miRNAs-155, -452, -224, and -210 in renal cell carcinoma [208]. Additionally, inhibiting miRNA-181 has been shown to cause Bcl-2 to be upregulated and Bax to be downregulated, protecting proximal tubular cells from damage from cisplatin [209].

The transforming growth factor-β (TGF-β)-induced miRNA-214 has been shown to upregulate the AKT pathway, inhibit monocyte and macrophage apoptosis, and promote renal interstitial fibrosis [210,211], whereas a report suggested that the function of miRNA-21 in promoting fibrosis in renal injury is independent of TGF-β signaling [212]. Nevertheless, miRNA-214 is upregulated in various models of AKI and renal fibrosis as well as in the monocytes of animals with CKD [212,213,214]. Renal fibrosis has been shown to be improved by the experimental antagonism of miRNA-214 [212]. Increased urinary miRNA-494 was associated with AKI, and this rise preceded changes in serum creatinine and urea nitrogen [215]. Since there were no variations in miRNA-494 serum concentrations, it was hypothesized that the miRNA-494 was of renal or urinary tract origin [215]. The same investigators then used a murine model of I/R injury to demonstrate an upregulation of miRNA-494 following injury, which inhibited the expression of activating transcription factor 3 (ATF3, a stress-response, reno-protective protein) and increased IL-6, MCP-1, and p-selectin [215]. In addition, miRNA-494 upregulated the expression of inflammatory cytokines and adhesion molecules through a nuclear factor-kappa B (NF-κB)-dependent pathway after kidney I/R injury. The same group discovered that miRNA-494 targeted numerous genes, including adiponectin receptor 2 (ADIPOR2), the B-cell lymphoma 2-like 11 apoptosis facilitator, and the IGF1 receptor (IGF1R), in the human Ingenuity Pathway Analysis or Target Scan System, in addition to ATF3. This would increase inflammation and cause more damage [215]. Mice receiving antisense-miRNA-494 before ischemia miRNA-494 had a clear pathophysiological effect in this AKI model because it ameliorated renal injury, as evaluated by serum creatinine, decreased proinflammatory cytokines, and caused the inhibition of caspase-3 apoptotic activity. Increased urinary miRNA-494 levels are reflective of AKI [215].

Bhatt et. al. showed that during renal I/R in mice and hypoxia in cultured kidney cells, miRNA-687 was noticeably elevated in the kidney [216]. They found that HIF-1 activated miRNA-687, which increased apoptosis by suppressing the phosphatase and tensin homolog (PTEN) [216]. Their animal studies have demonstrated that blocking miRNA-687 prevented kidney damage by maintaining PTEN expression, attenuated cell cycle activation, and reduced apoptosis, collectively suggesting that the HIF-1/miR-687/PTEN signaling pathway in I/R injury may be targeted for therapy [216].

**Table 2:** The miRNAs that are associated with AKI.

miRNA	Samples Type	Species	AKI Model/Population	Role in AKI	Reference
miRNA as biomarkers					
miRNA-10a	Plasma	Rat	I/R-induced kidney injury 12 h after reperfusion	Biomarker of diagnosis	[198]
	Serum	Human	Intensive care unit (ICU) and cardiac surgery (CS) patients	Biomarker of diagnosis	[201]
miRNA-23a	Serum	Human	Sepsis-induced acute kidney injury (AKI) (*n* = 6), sepsis-non-AKI (*n* = 6), and healthy volunteers (*n* = 3)	Biomarker of diagnosis	[217]
	Serum	Human	Acute myocardial infarction (AMI) AKI patients	Biomarker of diagnosis	[218]
miRNA-30 family	Plasma/Kidney tissue	Human/Rat	Contrast-induced nephropathy (CIN)	Biomarker of diagnosis	[219]
	Plasma	Human/Rat	Contrast-induced acute kidney injury (CI-AKI)	Biomarker of diagnosis	[220]
	Urine	Human/Rat	I/R-induced kidney injury	Biomarker of diagnosis	[221]
miRNA-126	Serum	Human	Intensive care units (ICU) and cardiac surgery (CS) patients	Biomarker of diagnosis	[201]
miRNA-127	Serum	Human	Intensive care units (ICU) and cardiac surgery (CS) patients	Biomarker of diagnosis	[201]
miRNA-146	Urine	Rat	Nephrotoxicity study in rats (cisplatin-induced kidney injury)	Biomarker of diagnosis	[193]
	Serum	Human	Intensive care units (ICU) and cardiac surgery (CS) patients	Biomarker of diagnosis	[201]
miRNA-192	Urine	Rat	Nephrotoxicity study in rats (cisplatin-induced kidney injury)	Biomarker of diagnosis	[193]
	Urine	Human/Rat	I/R-induced kidney injury	Biomarker of diagnosis	[221]
miRNA-210	Plasma	Human	Critically ill patients with acute kidney injury (AKI)	Biomarker of diagnosis	[222]
	Serum	Human	Intensive care units (ICU) and cardiac surgery (CS) patients	Biomarker of diagnosis	[201]
miRNA-494	Urine and kidney tissue	Human/Mouse	I/R-induced kidney injury	Biomarker of diagnosis	[215]
miRNA-489	Urine	Rat	Gentamicin-induced kidney injury	Biomarker of diagnosis	[223]
miRNA-668	Urine, serum, rat proximal tubular cells, kidney tissue	Human/Mouse	I/R-induced kidney injury	Biomarker of diagnosis	[224]

## 5. AKI, Inflammation, and H_2_S

Hydrogen sulfide (H_2_S), similar to nitric oxide and carbon monoxide, is a gasotransmitter. It is implicated in a number of inflammatory and vascular disorders that are associated with both pro- and anti-inflammatory signaling, including reducing the adhesion and rolling of circulating leukocytes in inflamed microvasculature, accelerating the resolution of experimental colitis, and enhancing gastric ulcer healing [102,225,226,227,228,229]. H_2_S can also modulate the immune response in the kidney, reduce inflammation, and promote tissue repair [230,231]. Additionally, it increases blood flow to the kidneys, aiding in their protection against injury [232,233]. Since then, it has been documented that a high amount of NaHS (1 mM) causes TNF-α and IL-1 release from IFN-γ primed U937 cells in a NF-kB-dependent fashion [234]. CSE, an enzyme that produces H_2_S, was found to be upregulated by LPS in a concentration-dependent manner in a study using primary macrophages (Mϕ) [235]. Conversely, H_2_S donors have been reported to reduce LPS-induced TNF-α release by microglia [21,27,236] and NF-kβ activation in RAW 264.7 macrophages [28]. Notably, mice deficient with the CBS gene suffer severe growth retardation and mortality within 5 weeks of birth [237]. Mice with CSE deficiency exhibit a milder phenotype, including age-related hypertension and sex-related hyperhomocysteinemia [238].

Numerous factors can lead to AKI and are categorized into three main groups: prerenal, caused by reduced kidney perfusion; renal, with obvious intrinsic kidney damage; and postrenal, caused by a blockage in the urinary tract [239]. However, renal I/R injury represents a leading cause of AKI [240]. I/R injury is a pathological condition that manifests in two steps: first, the blood supply to an organ is restricted, and then second, perfusion and re-oxygenation are restored [241]. One of the most vulnerable organs to I/R injury is the kidney [242]. Renal I/R is followed by a robust inflammatory response in which macrophages play a key role as mediators of inflammation and repair [243]. The two different functional subsets of macrophages are reported—classically activated macrophages (M1), which are mainly linked to pro-inflammatory immune responses, and alternatively activated macrophages (M2), which are primarily involved in tissue repair—and are used to classify macrophages, whether they are recruited or tissue-resident [244]. In an in vitro study using macrophages, lipopolysaccharide (LPS) stimulation decreased H_2_S production significantly [245]. Similarly, in an in vivo study on zymosan-induced peritonitis, granulocyte infiltration was associated with reduced H_2_S production [245]. When macrophages were treated with a slow-releasing H_2_S donor, GYY4137, it was found to suppress inflammatory cytokines and to promote anti-inflammatory chemokines in a concentration-dependent manner [246].

**Figure 6:**
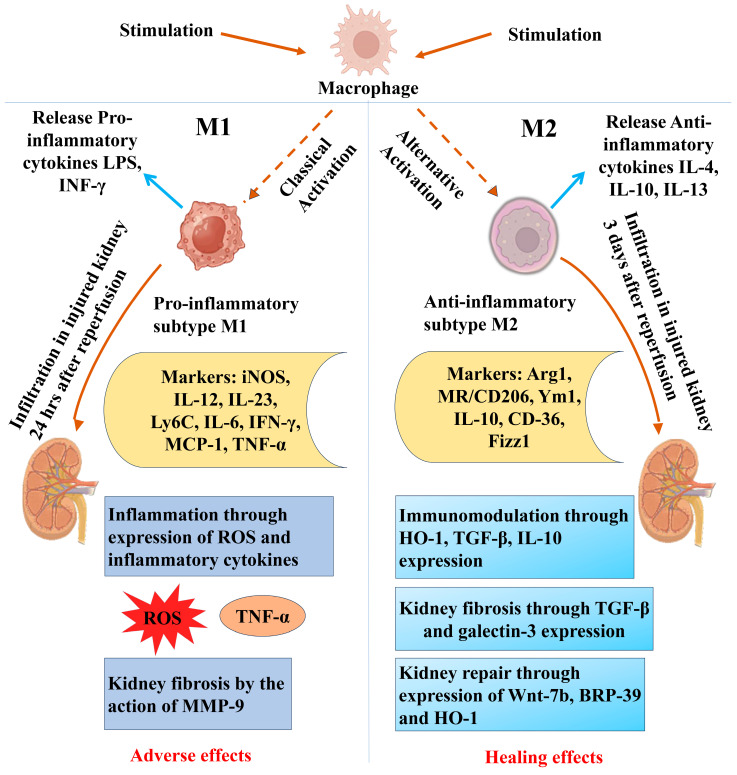
Two subtypes of macrophages (mφ)-M1 and M2 and their role in AKI. Distinct macrophage phenotypes are involved in renal injury and repair, especially in ischemia-reperfusion injury. Mφ are broadly classified into two subpopulations according to their phenotype and function. So-called “classically activated” pro-inflammatory macrophages (M1) infiltrate the kidney 24 h after reperfusion and contribute to kidney injury. M1 contributes to inflammation by the secretion of cytokines and reactive oxygen species (ROS). M1 also promotes kidney fibrosis through the release of MMP-9. In contrast, “Alternatively” activated anti-inflammatory macrophages (M2) are detected in the kidney 3 days after reperfusion, dampening renal inflammation and promoting tissue repair. M2 macrophages mediate kidney repair by the secretion of Wnt7b, BRP-39, and heme oxygenase-1 (HO-1). Additionally, galectin-3 and TGF-β, released by M2 macrophages, may induce renal fibrosis.

Resident renal mononuclear phagocytes may release proinflammatory cytokines, such as TNF-α, IL-1, and IL-6, and chemokines, such as CCL2, CCL5, CXCL10, and CXCL2, during renal I/R injury [247,248,249,250]. Therefore, a limited percentage of circulating Ly6C-monocytes and Ly6C+ monocytes invade the damaged kidney through a CCL2/CCR2 signaling cascade [247,249,251]. One hour after reperfusion, the wounded kidney experiences an increase in the influx of macrophages, which peaks at 24 h and lasts for 7 days [247]. The accumulation of macrophages in the postischemic kidney’s outer medulla is a hallmark of ongoing inflammation and tissue repair [252]. Infiltrating Ly6C+ monocytes may develop into classically activated macrophages, i.e., M1 macrophages, which express a proinflammatory phenotype in response to damage-associated molecular patterns (DAMPs) or proinflammatory mediators [240,247,252]. Exposure to lipopolysaccharide (LPS), IFN-γ, TNF-α, or GM-CSF induces M1 macrophages [252,253,254]. Neighboring immune cells, such as neutrophils, NK cells, and Th1/Th17 cells, release these inflammatory mediators in the renal interstitium [252]. Then, in a positive feedback loop, M1 macrophages release proinflammatory cytokines (such as TNF-α, IL-1, and IL-6) and ROS, which further increase I/R injury-induced AKI. Indeed, M1 macrophages play a role in both the activation of neutrophils and the triggering of apoptosis in epithelial cells [252,253,255]. Inducible nitric oxide synthase 2 (iNOS), IL-12, IL-23, and Ly6C are all highly expressed on these M1 macrophages, making them easy to spot [253,256]. The classical activation of the macrophage (M1) is shown on the left side of Figure 6.

M1 macrophages exhibit a proinflammatory phenotype with potent antimicrobial activity, and they stimulate or intensify CD4+ T cell polarization to the Th1 state by releasing IL-12 [257]. It is interesting to note that the liposomal clodronate (LC) suppression of kidney macrophages at the earliest stages of I/R injury lowers AKI and enhances renal healing, suggesting a crucial role for macrophages in I/R injury-induced AKI. The harmful function of M1 macrophages in ischemia AKI is further supported by the adoptive transfer of IFN-stimulated macrophages in LC-treated I/R injury mice, which worsens AKI [249,251,253,258,259,260,261,262,263]. Following the initial stages of I/R injury, Th2 and regulatory T (Tregs) cells are enlisted in the damaged renal tissue and produce significant amounts of IL-4, IL-10, and IL-13 [8,252,253,260,264,265,266,267,268,269].

In response to exposure to Th2-type cytokines (IL-4 and IL-13), macrophages change to the anti-inflammatory M2 phenotype, also known as alternatively activated macrophages, which is characterized by high Arg1 (arginase-1) expression, the mannose receptor (MR, also known as CD206), the chitinase-like protein (e.g., Ym1), the resistin-like protein (Fizz1), CD36 (fatty acid translocase), and IL-10 associated with the downregulated expression of pro-inflammatory markers, i.e., IL-12 and iNOS [8,252,253,254,255,257,267,268,269]. Notably, M2 macrophages can develop either directly from invading monocytes or through a transition from the M1 to M2 phenotype [253]. A tissue milieu that would encourage macrophage polarization towards the M2 profile is also created by the macrophage uptake of apoptotic cells that release large levels of anti-inflammatory cytokines, such as TGF-β and IL-10, in conjunction with a decrease in DAMPs [270,271,272]. An alternative activation of the macrophage (M2) is shown on the right side of Figure 6.

M2 macrophages exhibit an anti-inflammatory character and are essential for the immune response against parasites, wound healing, and fibrosis [273,274]. The three distinct subsets of M2 macrophages are M2a, which is triggered by exposure to IL-4 or IL-13, M2b, which is triggered by immune complexes like LPS or IL-1, and M2c, which is triggered by IL-10, TGF-β, or glucocorticoids. While M2c macrophages actively take part in tissue remodeling and exhibit regulatory features, M2a and M2b macrophages support a Th2 immune response, and although they have been identified in vitro, the subsets of macrophages do not accurately represent their activity in vivo [252,257]. These three distinct subgroups are recognized by distinctive gene signatures, which shed light on their role in the pathophysiology of I/R injury-induced AKI [275].

The opposing functions have been attributed to two subsets of macrophages, where M1 is pro-inflammatory and M2 is a regulator of tissue repair [244]. We have shown that H_2_S expedited macrophage differentiation from M1 to M2 in AKI, both in the young and old kidneys with a greater extent in the young kidney [232]. The above information suggests an intricate mechanism of AKI and repair process involving macrophages in the inflammatory and anti-inflammatory response, and H_2_S is a regulator of that process. It is therefore important to evaluate H_2_S production and availability in the kidney under pathological conditions, such as in I/R for effective diagnosis and therapeutic strategies, particularly in aging AKI.

## 6. AKI and Matrix Biology: Role of EMMPRIN, Meprin-A, and MMPs

The extracellular matrix metalloproteinase inducer (EMMPRIN), also known as CD147 or Basigin, belongs to the immunoglobulin (Ig) superfamily. It is abundant on the surface of tumor cells and stimulates the production of several matrix metalloproteinases (MMPs) by adjacent stromal cells. It is also widely expressed in numerous cell types [154,276,277]. It acts as a cell surface receptor for a variety of ligands to activate a number of MMPs, which are involved in a number of processes, such as cell disruption, inflammation, tissue healing, and remodeling [277].

Notably, MMPs are a family of enzymes that can degrade a wide variety of extracellular protein (ECM) proteins [278]. There are over 20 different MMPs, and they are classified into four groups: collagenases, gelatinases, stromelysins, and membrane-type MMPs [278]. Anti-EMMPRIN antibody therapy is cardioprotective, while EMMPRIN-induced MMP-9 production has been shown to promote myocardial I/R injury [279].

Meprins are astacin family zinc-dependent metalloproteinases that were first discovered and characterized in the mouse’s brush-border membranes [280], rat kidneys [281], and human intestines [282]. In addition to EMMPRIN, metallo-endopeptidase Meprin-A also activates MMP-9 [283]. Within the kidney, Meprin-A localizes to the membranes of the brush-border of proximal tubules [284]. Numerous studies have shown that Meprin-A plays a significant part in AKI, which involves ECM degradation leading to remodeling [285,286]. Others have shown that elevated MMP-9 expression and activity cause extensive damage to the tubules, glomeruli, and interstitial regions [287,288,289]. These studies suggest that the induction of EMMPRIN, Meprin-A, and MMP-9 is intricately associated with AKI in the young. However, the extent of their dysregulation in I/R-induced AKI in aging was not clear until we recently showed that EMMPRIN, Meprin-A, and MMP-9 are also upregulated in aging AKI [232]. The extent of their upregulation was higher than young AKI, and H_2_S mitigated their expression, both in young and old kidneys, suggesting that H_2_S plays a crucial role in their regulation during AKI pathology. Additionally, it has been reported that hyperhomocysteinemia (HHcy), which causes a decrease in H_2_S levels, increases the M1 macrophage phenotype, and this effect was partially attributed to EMMPRIN induction [290].

MMPs represent inflammatory cell-derived cytokines, another important group of fibrosis-related inflammatory cytokines [291,292,293]. MMP-2 and -9 were both associated with the progression of renal fibrosis [291,293,294]. MMPs suppressed fibrosis through the degradation of ECM components [292,295]. In NaHS treatment, the renal expression of MMP-2 and MMP-9 in diabetic kidney disease rats was downregulated [293,296,297]. Our results indicated that diabetic kidneys express high levels of MMP-9 with increased activity, resulting in the downregulation of H_2_S production by diminishing CBS and CSE enzymes. In the downstream pathway, low levels of H_2_S induced NMDA-R1 and elevated Cxs-40 and -43. When considered collectively, these findings imply that H_2_S plays a major role as a mediator in diabetic kidney remodeling [298]. It has also been suggested that GYY4137 therapy stabilizes MMP-9, MMP-13, and MMP-14 levels and lowers ROS-induced kidney fibrosis by increasing microRNA-194 [299].

In addition to MMP-9, other MMPs such as MMP-1, -2, -3, -8, and -13 are also related to ECM remodeling during progressive renal diseases [300,301]. Collagens I and III are degraded by the interstitial collagenases MMP-1, -8, and -13 [302], whereas MMP-3 favors ECM proteins, particularly fibronectin [303]. The role of MMP-7 in the progression of tubulointerstitial fibrosis is well known [304], and MMP-12 in podocytes is implicated in glomerulonephritis [305]. Interestingly, MMP activity is regulated by the tissue inhibitors of metalloproteinases (TIMPs), and TIMP-1 inactivates most MMPs [306,307,308].

So, we can conclude that H_2_S has the therapeutic potential to reduce the remodeling of the kidneys associated with diabetes while also suppressing the inflammatory response. Further research is still needed to fully understand how H_2_S modulation affects the inflammation of the AKI.

## 7. ECM and Vascular Dysfunction in Renal Aging and AKI

The endothelium extensively covers the inner surface of the cardiovascular system. Its polarized architecture in blood vessels, heterogeneity in morphology, structure, and gene expression profile at various locations of different blood vessel types demonstrate its functional importance [309]. Vascular inflammation normally begins and spreads in the endothelium. H_2_S suppresses vascular inflammation [102,309] by activating K_ATP_ and BKCa channels as well as HO-1 expression, inhibiting p38, and the nuclear factor kappa-light-chain-enhancer of activated B cells (NF-κB) [310,311]. Additionally, H_2_S reduces the amount of reactive oxygen species (ROS) in endothelial cells [62,312,313,314], which is accomplished in part by scavenging ROS [105,315] and in part by improving antioxidant defense mechanisms.

I/R injury is defined as cellular damage by ischemia followed by reperfusion of formerly viable ischemic tissues [316]. Endothelial injury is the hallmark of I/R injury [317]. Ischemia promotes the expression of pro-inflammatory molecules and cytokines in the endothelium while decreasing the production of beneficial molecules, including nitric oxide, thrombomodulin, and prostacyclin [316]. In addition, it may decrease H_2_S production as there is evidence that CBS, CSE, and 3-MST are also present in the vascular endothelium [23,318,319,320,321].

Pathological situations in the adult organism, such as injury, inflammation, or aging, can activate endothelial-to-mesenchymal transition (EndoMT) and cause the fibrosis of the affected organs [322]. In addition to renal fibrosis, the EndoMT has been postulated to play a role in the initiation and advancement of cardiac, pulmonary, hepatic, corneal, intestinal, unilateral ureteral obstruction (UUO), diabetes, and wound healing [323,324,325,326,327].

In a groundbreaking study, Zeisberg et al. used three mouse models of renal fibrosis—UUO, a model for studying progressive tubulointerstitial fibrosis, streptozotocin (STZ)-induced DN, and alpha 3 chain of collagen type 4 (COL4A3) knockout mice—to confirm first the role of EndoMT in renal fibrosis [327]. A considerable fraction of myofibroblasts co-expressed the endothelium marker CD31, also referred to as platelet endothelial cell adhesion molecule-1 (PECAM-1), in all three models. Additionally, they co-expressed the markers for myofibroblasts, α-smooth muscle actin (α-SMA), and fibroblast-specific protein-1 (FSP-1, equivalent to S100A4) [327]. During the onset and development of pathological fibrosis, an excessive and uncontrolled production of ECM is caused by the accumulation of many myofibroblasts [323]. Emerging evidence also suggests that the vascular endothelium is one of the sources of myofibroblasts. Endothelial cells become myofibroblasts when tissue-infiltrating chronic inflammatory cells, such as macrophages and lymphocytes, produce TGF-β. The endothelial-derived mesenchymal cells then go into the interstitium and take part in tissue fibrosis [323]. By examining phosphorylated Smad3, the TGF-β pathway’s activation was determined, and TGF-β blocking experiments made use of a particular Smad3 inhibitor (SIS3). These findings showed that Smad3 activation was necessary for advanced glycation end-products (AGEs) to activate EndoMT in microvascular endothelial cells in vitro and in transgenic mice in vivo [323,328]. Scientific evidence also shows that EndoMT is a unique mechanism that contributes to the formation of fibrosis in DN, and it has been proposed that blocking EndoMT using inhibitors of the TGF-β pathway may offer a new strategy to slow the evolution of DN and other fibrotic processes [323]. Lineage tracing indicated that about 30–50% of kidney fibroblasts in UUO nephropathy, DN, and Alport renal disease models expressed endothelial markers, indicating a large contribution of endothelial cells to fibroblasts through phenotypic change [327].

Collagen IV is found in the glomerular basement membrane (GBM), which serves as a structural and functional barrier between the vascular system and the urinary space [329]. Further, the components of the renal interstitial matrix are collagen types I, III, V, VI, VII, and XV, and collagen types I and III are reported to be deposited in the early stages of renal fibrosis [330,331,332].

In an article, Patschan et. al. postulated that mid-term prognosis in AKI depends on, in part, the presence of interstitial fibrosis by EndoMT. They also found that treatment with early endothelial progenitor cells (eEPCs) can reduce postischemic EndoMT and fibrosis in young AKI mice [333]. Additionally, we have demonstrated that during I/R injury, the aging kidney showed increased EndoMT and fibrosis compared to the young kidney [232]. After receiving treatment with GYY4137, an H_2_S donor, the aging kidney’s vascular density, blood flow, and renal function were all enhanced. We concluded that H_2_S can rescue kidney dysfunction in aging mice due to the I/R injury that is related to EndoMT and fibrosis [232]. Nevertheless, further and broader studies are needed to confirm these initial findings for a viable approach for aged patients sustaining AKI.

## 8. Current Treatment Modalities and Future Strategies

The therapeutic strategies for AKI based on the KDIGO guidelines and bundles of care are limited and mostly supportive [334]. There is not any approved drug that has been designated to treat, prevent, or speed up the healing process from AKI. The primary goal of current interventions is to stop additional declines in renal function [335]. The clinical approach should begin with hemodynamic stabilization, the early detection of AKI consequences, understanding of its etiology, and management [336]. Because autoregulation mechanisms are compromised in AKI, hemodynamic stability is crucial [337].

To prevent negative effects, special attention should be given to drugs with renal toxicity, which should be either stopped or the dose adjusted based on renal function [336,338]. Furthermore, it is essential to start antibiotics as soon as possible in septic patients [339].

**Figure 7:**
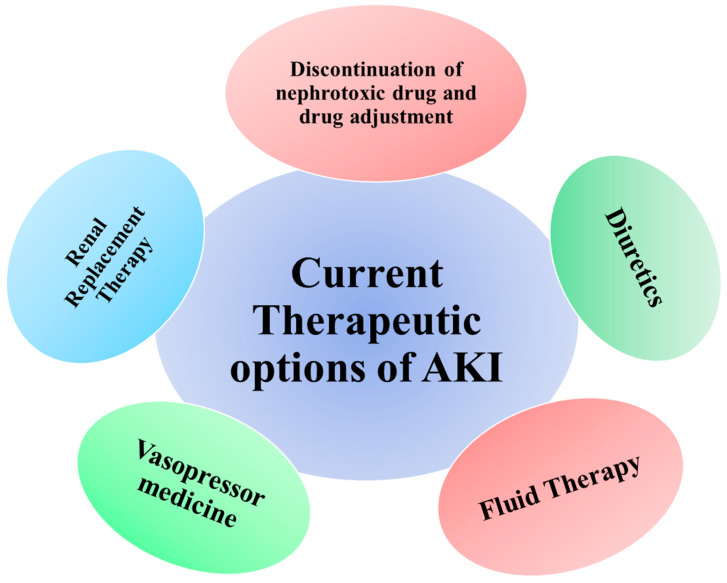
Schematic diagram showing some of the current therapeutic options of AKI.

Finally, when treating an AKI patient, it is critical to quickly detect and address additional issues, such as hyperkalemia, metabolic acidosis, anemia, and fluid overload [334]. It is also advised to begin stress-ulcer prophylaxis and prevent infection during AKI [334]. Figure 7 summarizes current therapeutic modalities in AKI.

### 8.1. Fluid Therapy

In individuals with AKI, intravascular hypovolemia is a serious consequence. It is corrected by individualized fluid therapy, whose main aim is to increase intravascular blood flow and increase cardiac output [337]. Fluids of fluid therapy are broadly classified into colloid (albumin, starch) and crystalloid (saline, Ringer’s lactate, or PlasmaLyte) [337,340].

Fluid therapy should be personalized based on the patient’s physiological parameters, underlying diagnosis, trajectories, and the overall risks and benefits of fluids [337]. Although the ideal fluid type is still uncertain, starches should be avoided because trials have reported that starches can worsen the incidence of AKI due to osmotic nephrosis with proximal vacuolization and swelling [341].

### 8.2. Vasopressor Medicine

The median blood pressure of an AKI patient must be greater than 65 mmHg, which can maintain accurate renal perfusion. After fluid therapy or volume resuscitation, a vasopressor drug should be given to ensure enough renal perfusion to prevent a positive fluid balance [334,342,343,344]. The recommended first-line vasopressor in a vasodilatory state is noradrenaline [345,346]. It increases perfusion pressure and maintains circulatory flow. The lowest dose of noradrenaline is recommended because higher doses can cause decreased blood flow through excessive vasoconstriction [347].

Vasopressin and Terlipressin are two effective alternatives for rising blood pressure, although their comparison with noradrenaline has not been demonstrated yet [348,349,350,351]. Angiotensin II has shown promising results on patient outcomes in recent studies, namely, by improving survival and renal function recovery [352]. However, further research is still needed before angiotensin II should be used regularly.

### 8.3. Diuretics

To achieve electrolyte balance and manage fluid overload in AKI, diuretics are used [334,353]. It was previously thought that loop diuretics might protect the loop of Henle from ischemia by decreasing its workload [353]. It has never been proven that furosemide has any clinical advantages in preventing AKI, reducing the requirement for renal replacement therapy (RRT), promoting renal recovery, or lowering in-hospital mortality [354,355,356]. It is important to note that certain studies have associated the usage of loop diuretics with a higher risk of death, which might be related to the delay in appropriate RRT start [353,356,357]. The disadvantages related to diuretics include how high doses of loop diuretics can cause ototoxicity [353]. As a consequence, the KDIGO guideline does not recommend the use of diuretics as an AKI treatment [334].

### 8.4. Drug-Induced AKI

Drug toxicity is the major cause of AKI and has been associated with 20–40% of AKI cases, and it goes up to 60% in elderly patients [358,359]. Drug nephrotoxicity is diverse and varies with many classes of drugs. Drugs can cause direct kidney damage, such as tubular injury, interstitial nephritis, glomerular injury, obstructive nephropathy, and indirect harm by reducing renal blood flow [360]. Moreover, drug-induced nephrotoxicity arises from innate drug toxicity, changed renal hemodynamic characters, prior renal issues, and altered drug pharmacokinetics in critical illness [360,361]. Medications and chemicals commonly linked to nephrotoxicity include calcineurin inhibitors, which increase the chance of developing prerenal AKI, renin–angiotensin–aldosterone system blockers, high-dose systemic vasoconstrictors, and nonsteroidal anti-inflammatory medicines (NSAIDs) [358]. Aminoglycosides, vancomycin, radiocontrast agents, cisplatin, amphotericin B, foscarnet, and osmotically active substances are associated with acute tubular necrosis (ATN), which is dose-dependent and the common form of drug-induced AKI in hospitalized patients [358,362]. Patients treated with acyclovir, ciprofloxacin, methotrexate, and sulfa-based medicines may develop AKI as a result of crystal-induced luminal blockage [362].

To reduce toxicity, medication prescriptions must be properly thought out. The KDIGO recommendations urge early drug withdrawal of potentially nephrotoxic medications, avoiding radiocontrast and other potentially harmful pharmaceuticals, and drug dose monitoring [334].

### 8.5. Renal Replacement Therapy

Anuria, severe/refractory metabolic acidosis, severe/refractory hyperkalemia, severe azotemia, refractory volume overload, or clinical effects of uremia such as encephalopathy, pericarditis, or neuropathy are conventional criteria for initiating RRT in AKI [363,364]. RRT is necessary to preserve the homeostasis of volume, electrolytes, acid-base, and uremic solutes in AKI patients. Additionally, it is hypothesized that RRT can reduce inflammation, which may be important for septic patients; however, this is still unknown [363,365]. The disadvantages of this therapy are that RRT necessitates the insertion of a central venous dialysis catheter, the contact of blood with an extracorporeal circuit, and anticoagulation. It can also be linked to hemodynamic instability, which may delay kidney recovery [363,365]. Although there has not been any proof that the risk classification of patients for AKI can help with long-term renal outcomes, it can enable the use of appropriate treatments and reduce the incidence of AKI [366,367,368].

Perna et. al. showed that the transcriptional dysregulation of genes encoding H_2_S-producing enzymes resulted in a decrease in H_2_S levels in hemodialysis patients [369]. In a separate communication, they also reported interesting findings on H_2_S levels before and after single standard 4 h hemodialysis sessions in 131 patients, which were conducted in two dialysis sessions separated by one month [370]. Their results indicated that plasma H_2_S was significantly increased and homocysteine (Hcy) levels were significantly decreased after dialysis compared to before dialysis, suggesting that hemodialysis is effective in reestablishing the altered H_2_S homeostasis in these patients. They also interpreted through reaction stoichiometry analysis that an improved Hcy metabolism occurred through H_2_S formation during dialysis [370]. These results strongly suggest that RRT is effective in maintaining H_2_S homeostasis in kidney failure patients, which can possibly be applied to AKI patients as well.

## 9. Summary and Discussion

Acute kidney injury (AKI) is very common and significantly reduces the normal function of the kidney. The mortality of AKI remains high due to the lack of early diagnosis and cause-specific treatment. Thus, it is a significant burden for both the patient and society. A specific treatment has not yet been developed for AKI probably due to the complex nature of the condition and our inability to recognize AKI phenotypes at this time.

I/R injury is one of the main causes of AKI. AKI prevention and early detection are our main options for lowering the incidence and harmful effects of AKI because there are no viable treatment therapies for established AKI and because of its high morbidity and mortality. Conversely, one could counter that risk assessment is ineffective since it is not evident which interventions are beneficial to use and which ones should be applied to high-risk individuals.

H_2_S has beneficial effects on aging in the body by acting as an antioxidant against oxidative species such as ROS and RNS. H_2_S is generated by several enzymes, i.e., CBS, CSE, CAT, and 3MST, and protects cells from damage, reduces inflammation, and improves mitochondrial function. However, more research is needed to confirm these findings and to determine how H_2_S could be used to slow down the aging process in humans.

Following renal I/R injury, the miRNA expression profile was studied, and it shows a survival response. In fact, the majority of miRNAs that were discovered seem to have differential expression, possibly suppressing the damage response by controlling apoptosis, proliferation, and inflammation. These findings may help us identify novel pathways that can be targeted to prevent or reduce renal injury. If the miRNAs found to be expressed differently can be shown to play a role in these processes in vivo, they may represent new biomarkers of renal injury.

Leukocyte adhesion to the endothelium and endothelial permeability are both regulated by H S. The anti-inflammatory H_2_S effects seem to be mediated through activating K_ATP_ channels; thus, these K_ATP_ channels are potential targets for anti-inflammatory therapies in AKI among aged patients. The early influx of macrophages promotes a proinflammatory state that amplifies tissue injuries. In response, M1-recruited monocytes or tissue-resident macrophages change into M2 macrophages in response to local cues, which help to promote tissue repair and inhibit the renal inflammatory response. The depletion of macrophages 3 days after I/R injury slows the rebuilding of renal tissue, but the depletion of macrophages before I/R injury lessens renal insult. More specifically, the information that is currently available strongly suggests that H_2_S may develop into the next effective preventative and therapeutic agent for preventing and treating aging-related illnesses and age-associated diseases, and this should be studied in different aged AKI models.

## 10. Future Perspective

The management of AKI is poor in underdeveloped countries due to high costs and late diagnosis. In addition, currently available therapies have major side effects. Nevertheless, a range of specific treatment strategies is emerging that may have an influence on the prognosis of patients with AKI, thanks to recent advancements in the knowledge of its pathophysiology and advancements in trial design. Additional biomarkers could be used to define AKI more precisely, leading to earlier identification [371,372]. While the research on H_2_S and aging is still in its early stages with knowledge gaps, it is a promising area of research. If H_2_S has beneficial effects on renal aging, it could potentially be used to develop new therapies for delaying the onset of age-related diseases and extending lifespans. On the other hand, current information demonstrates that miRNAs have a potential role in I/R injury-induced AKI; however, the dysregulation of miRNAs and their functional relevance in aging kidneys have yet to be fully deciphered.

## Data Availability

Not applicable.

## Abbreviations

3-MST, 3-mercaptopyruvate sulfur transferase; AKI, acute kidney injury; AKT, protein kinase B; ATF3, activating transcription factor 3; ATN, acute tubular necrosis; BIM, a pro-apoptotic BCL2-family protein; CBS, cystathionine β-synthase; CKD, chronic kidney disease; CSE, cystathionine γ-lyase; DAO, D-amino acid oxidase; DAMPs, damage-associated molecular patterns; DN, diabetic nephropathy; ECM, extracellular protein; EMMPRIN, extracellular matrix metalloproteinase inducer; EndoMT, endothelial-to-mesenchymal transition; GBM, glomerular basement membrane; GFR, glomerular filtration rate; H2S, hydrogen sulfide; I/R, ischemia-reperfusion; IL, interleukin; miRNA, micro RNA; MCP-1, monocyte chemoattractant protein-1; PTEN, phosphatase and tensin homolog; RBF, renal blood flow; ROS, reactive oxygen species; RRT, renal replacement therapy; STZ, streptozotocin; TECs, tubular epithelial cells; TGF-β, transforming growth factor-β; TNF, tumor necrosis factor; TST, thiosulfate transferase; UUO, unilateral ureteral obstruction.
